# Influenza Virus-Induced Robust Expression of SOCS3 Contributes to Excessive Production of IL-6

**DOI:** 10.3389/fimmu.2019.01843

**Published:** 2019-08-16

**Authors:** Shasha Liu, Ruoxiang Yan, Biao Chen, Qidong Pan, Yuhai Chen, Jinxuan Hong, Lianfeng Zhang, Wenjun Liu, Song Wang, Ji-Long Chen

**Affiliations:** ^1^CAS Key Laboratory of Pathogenic Microbiology and Immunology, Institute of Microbiology, Chinese Academy of Sciences, Beijing, China; ^2^College of Animal Sciences, Fujian Agriculture and Forestry University, Fuzhou, China; ^3^College of Life Sciences, University of Chinese Academy of Sciences, Beijing, China; ^4^Institute of Laboratory Animal Science, Chinese Academy of Medical Sciences & Comparative Medical Center, Beijing, China

**Keywords:** immune response, SOCS3, IL-6, inflammation, influenza virus

## Abstract

Influenza A virus (IAV) remains a major public health threat in the world, as indicated by the severe pneumonia caused by its infection annually. Interleukin-6 (IL-6) involved excessive inflammatory response to IAV infection profoundly contributes to the virus pathogenesis. However, the precise mechanisms underlying such a response are poorly understood. Here we found from both *in vivo* and *in vitro* studies that IAV not only induced a surge of IL-6 release, but also greatly upregulated expression of suppressor of cytokine signaling-3 (SOCS3), the potent suppressor of IL-6-associated signal transducer and activator of transcription 3 (STAT3) signaling. Interestingly, there existed a cytokine-independent mechanism of the robust induction of SOCS3 by IAV at early stages of the infection. Furthermore, we employed SOCS3-knockdown transgenic mice (TG), and surprisingly observed from virus challenge experiments using these mice that disruption of SOCS3 expression provided significant protection against IAV infection, as evidenced by attenuated acute lung injury, a higher survival rate of infected animals and lower viral load in infected tissues as compared with those of wild-type littermates under the same condition. The activity of nuclear factor-kappa B (NFκB) and the expression of its target gene IL-6 were suppressed in SOCS3-knockdown A549 cells and the TG mice after infection with IAV. Moreover, we defined that enhanced STAT3 activity caused by SOCS3 silencing was important for the regulation of NFκB and IL-6. These findings establish a critical role for IL-6-STAT3-SOCS3 axis in the pathogenesis of IAV and suggest that influenza virus may have evolved a strategy to circumvent IL-6/STAT3-mediated immune response through upregulating SOCS3.

## Introduction

Upon the recognition of viral pathogen-associated molecular patterns (PAMPs) by pattern recognition receptors (PRRs), such as retinoic acid-inducible gene I-like receptors (RLRs), toll-like receptors (TLRs), C-type lectin receptors (CLRs) or NOD-like receptors (NLRs) (1, 2), various inflammatory cytokines including interleukin (IL) 6 (IL-6), IL-1β, and tumor necrosis factor alpha (TNFα) are produced in the host in response to the infection (3–5). Although these cytokines play critical antiviral roles in the first wave of host immune response (6–8), the excessively produced pro-inflammatory cytokines contribute to increased disease severity. For example, IL-6, an important marker and mediator of ongoing inflammation, is expressed by innate immune cells (macrophage and dendritic cells) or non-immune cells (epithelial cells and fibroblasts) during influenza A virus (IAV) infection. As a multi-functional cytokine, IL-6 has been shown to be required for the virus clearance and mouse survival through modulating neutrophil release and recruitment, and pivotal for the antibody production by promoting the differentiation of B cells (6, 7). Further, IL-6 prevents tissue destruction through elevating the secretion of cytoprotective cytokine IL-22 (9, 10). However, aberrant and excessive production of IL-6 is associated with highly virulent IAV infection, which leads to severe disease progression (11). In addition, dysregulation of IL-6 expression is observed in various chronic inflammation and autoimmunity (12). Within the complex regulatory network of IL-6, IL-6-activated signal transducer and activator of transcription 3 (STAT3) and the primary inhibitor of IL-6/STAT3 signaling, suppressor of cytokine signaling 3 (SOCS3), are essential for the function of IL-6.

STAT3 is a signaling mediator of IL-6 and other cytokines or growth factors such as IL-10, IL-21, interferon-gamma (IFN-γ), leukemia inhibitory factor (LIF), epidermal growth factor (EGF), and granulocyte colony-stimulating factor (G-CSF) (13–15). As a transcription factor, STAT3 has a wide variety of functions in mammalian cells (16–18). Following the engagement of IL-6 with the receptor complex containing gp130 and IL-6R/sIL-6R, STAT3 is phosphorylated by the JAK kinases (19). The IL-6-activated STAT3 promotes the transcription of various target genes, including pro-inflammatory genes, anti-inflammatory genes and IFN-inducible genes, which play central roles in the development, recruitment and anti-apoptosis of macrophages, T cells, and B cells (19, 20). Nevertheless, SOCS3 specifically inhibits the activity of STAT3 to attenuate IL-6 action and is a key regulator for controlling the duration of IL-6 signaling and STAT3 activation (20).

SOCS proteins inhibit STAT3 by competitively binding with phosphorylated tyrosine residues of JAKs, facilitating the receptor-JAK-STAT complex ubiquitination and degradation and inhibiting the JAK activities (21). In the absence of SOCS3, IL-6 acts to suppress TNF but induce an IFN-regulated transcriptional response in macrophages (20). This is consistent with the observations that mice lacking SOCS3 have a considerably higher survival rate after lipopolysaccharide challenge or exhibit a lower viral load after virus infection (9, 10, 20, 22). Moreover, the IL-7-mediated antiviral effects are dependent on the increased endogenous IL-6 and the substantial repression of SOCS3 (9). However, the relationship between IL-6, STAT3, and SOCS3 and their functions in antiviral response remain to be defined.

In addition, several lines of evidence have suggested that SOCS3 plays important roles in immune and inflammatory responses based on the altered expression of SOCS3 after viral infection (23). For example, it has been observed that SOCS3 is significantly upregulated by infections with IAV (24, 25), respiratory syncytial virus (RSV) (26), SARS coronavirus (SARS-CoV) (26), Herpes simplex virus type 1 (HSV-1) (27), or human immunodeficiency virus (HIV) (28). High levels of SOCS3 are also shown in liver specimens from patients with Hepatitis C virus (HCV) genotype 1 infection (29) and chronic Hepatitis B virus (HBV) infection (30). During viral infection, the expression of SOCS3 could be induced by viral protein or RNA (25, 28, 31), such as HIV-1 regulatory protein Tat, HCV core protein, RSV protein NS1, or 5′ triphosphate RNA of IAV, and by host cytokines, such as TNFα, IL-6, or IFNγ (7, 32–34), through various activating transcription factors, including nuclear factor-kappa B (NFκB), STAT3, and STAT1 (7, 32, 33). These studies indicate that tight control of SOCS3 expression via some feedback pathways is critically associated with maintaining the appropriate balance of host antiviral response, and thus dysregulation of SOCS3 may contribute to viral pathogenesis.

In this study, we found that the expression of both IL-6 and SOCS3 was greatly induced *in vitro* and *in vivo* during the IAV infection. Interestingly, IAV-induced early expression of SOCS3 was independent of IL-6 and type I IFNs. We observed that disruption of SOCS3 expression significantly inhibited the virus replication and increased the survival rate of SOCS3-knockdown transgenic (TG) mice after IAV challenge. Furthermore, our experiments demonstrated that silencing SOCS3 resulted in an increase in STAT3 activity, which decreased the activation of NFκB and thereby impaired the expression of IL-6. Therefore, the suppression of IL-6/STAT3 signaling by elevated SOCS3 induced by IAV might contribute to excessive production of IL-6 during the virus infection. These observations provide important evidence that the IAV-induced SOCS3 is critically involved in regulation of IL-6-mediated inflammatory response to the virus infection.

## Materials and Methods

### Ethics Statement

Mice were bred and housed in a colony room at the Institute of Microbiology, Chinese Academy of Sciences. The humidity (50–70%) and temperature (21–24°C) were controlled. The room was maintained on a 12:12 light: dark cycle and water was available. The animals lived in autoclaved individual ventilated cages (IVC) in groups of up to five mice with same sex each cage. The animal experimental protocol used in this study was in accordance with the guidelines contained in the International Guiding Principles for Biomedical Research Involving Animals (CIOMS & ICLAS, 2019) and was approved by the Research Ethics Committee of Institute of Microbiology, Chinese Academy of Sciences (permit number APIMCAS2017045). All mouse experimental procedures were performed in accordance with the Regulations for the Administration of Affairs Concerning Experimental Animals approved by the State Council of the People's Republic of China.

### Cell, Virus, and Viral Infection

293T (American Type Culture Collection (ATCC) CRL-11268), A549 (ATCC CCL-185), MDCK (ATCC CRL-2935), and RAW264.7 (ATCC TIB-71) cells were maintained in Dulbecco's modified Eagle's medium (DMEM) (Gibco-BRL, Gaithersburg, MD, USA), or THP1 (ATCC TIB-202) cells in RPMI1640 (Gibco-BRL, Gaithersburg, MD, USA), containing 10% fetal bovine serum (FBS) (Gibco-BRL, Gaithersburg, MD, USA) supplemented with penicillin (100 U/mL) and streptomycin (100 μg/mL). IAV H1N1 strains including A/WSN/33 (WSN), A/PR/8/34 wild type (PR8) and A/CA/04/09 (CA04) were propagated in specific-pathogen-free (SPF) chicken embryo as previously described (35). For viral infection, cells were infected with virus at the indicated multiplicity of infection (MOI). After adsorption for 45 min at 37°C, the cells were washed with phosphate-buffered saline (PBS) and cultured in DMEM with 2 μg/mL trypsin for indicated time.

### cDNA Microarray and Data Analysis

cDNA microarray experiments were performed using mouse 4 × 180 K gene expression microarray (Agilent Mouse lncRNA 049801). The lungs of mice were prepared using Trizol reagent (Life Technology, Carlsbad, CA, USA). Total RNAs were from three independent groups of WSN-infected mice (5 × 10^4^ plaque forming unit (PFU), 24 hpi) or uninfected control mice. cDNA synthesis, labeling, hybridization, and data analysis were carried out as previously described (36). Expression data were normalized through quantile normalization.

### Antibodies, Chemicals, and Plasmids

The following antibodies were used for Western blotting in this study: anti-STAT3 (124H6) (1:1,000), anti-phospho-STAT3 (Y705) (1:1,000), anti-β-actin (1:3,000) and anti-flag (1:800) (Cell signaling Technology, Danvers, MA, USA); anti-IKB-α (1:2,000) (Santa Cruz Biotechnology, Santa Cruz, CA, USA); anti-influenza A virus NP (1:10,000) polyclonal antibody was obtained by immunizing rabbits with GST-tagged NP protein. The STAT3 inhibitor S3I-201 was purchased from Selleck Chemicals (Houston, TX, USA). The protein synthesis inhibitor cycloheximide (CHX) was purchased from Sigma-Aldrich (St. Louis, MO, USA) and used at the concentration of 1 μg/mL during the pretreatment (30 min) of cells and viral infection (as indicated). Plasmids pRC-CMV-STAT3-WT, pRC-CMV-STAT3-D661V (661 aspartic acid of STAT3 was substituted by valine) and pRC-CMV-STAT3-Y640F (640 tyrosine of STAT3 was substituted by phenylalanine) were constructed by inserting the cDNA coding STAT3-WT, STAT3-D661V and STAT3-Y640F into the Not I/Sal I site of retroviral vector pMSCV-IRES-GFP (pMIG) (37).

### Western Blotting and Immunofluorescence

Cell lysates were prepared, and Western blotting was performed as previously described (38). Briefly, samples were separated on SDS-polyacrylamide gel, transferred onto a nitrocellulose membrane, and probed with antibodies as indicated. For immunofluorescence, A549 cells were infected with A/WSN/33 virus (MOI = 0.5) for 15 h and fixed with 4% paraformaldehyde. Then the samples were incubated with anti-NFκB p65 (Santa Cruz Biotechnology, Santa Cruz, CA, USA) and Alexa Fluor-labeled anti-mouse secondary antibody (Jackson Immuno Research, PA, USA), and counter-stained with 4′-6-diamidino-2-phenylindole (DAPI) (Keygene, Jiangsu, China). Imaging was performed using a FluoView (FV1000) confocal laser scanning microscope (Olympus Optical, Japan).

### RNA Extraction, RT-PCR, and Quantitative RT-PCR

Total RNA was extracted from cells or tissues using TRIzol reagent (Invitrogen, Carlsbad, CA, USA) and treated with DNase I (TaKaRa, Tokyo, Japan). cDNA was synthesized using 4 μg of total RNA and GoScript reverse transcriptase (Promega, Madison, WI, USA), followed by PCR using rTaq DNA polymerase or quantitative RT-PCR (RT-qPCR) using SYBR Premix Ex Taq II (TaKaRa, Tokyo, Japan) with the primers shown in Table S1. Actin was chosen as a reference gene for internal standardization.

### RNA Interference and Generation of Stable Cell Lines

Short hairpin RNA (shRNA)-based knockdown cell lines were generated by infection of A549 with lentiviruses expressing specific shRNAs in pSIH-H1-GFP vector as described previously (36). The sequences used in the shRNAs targeting specific genes were as follows: sh-STAT3 (human) 5′-GCAGCAGCTGAACAACATG-3′, sh-SOCS3 (human) 5′-CCACCTGGACTCCTATGAGAA-3′, sh-IL-6Rα (human) 5′-GGAAGACAATGCC ACTGTTCA-3′, and sh-gp130 (human) 5′-GCTCACTTGCAACATTCTTAC-3′. Cell lines stably expressing STAT3-WT, STAT3-D661V, and STAT3-Y640F or empty vector (EV) were generated by infecting the cells with retroviruses encoding these genes in pMIG vector as previously described.

### Plaque Forming Assay and Hemagglutinin Assay

Plaque forming assay and hemagglutinin (HA) assay were performed as previously described (36). Briefly, for plaque forming assay, MDCK cells were incubated with serial dilutions of the cell culture supernatants for 1 h, washed with PBS and overlaid with DMEM containing low-melting-point agarose (Promega, Madison, WI, USA) and 2 μg/mL tolylsulfonyl phenylalanyl chloromethyl ketone (TPCK)-treated trypsin (Sigma-Aldrich, St. Louis, MO, USA). After incubation for 72 h, plaques were stained and counted. For hemagglutinin assay, the supernatants were serially 2-fold diluted with PBS and mixed with an equal volume of 0.5% chicken erythrocytes. Then, viral titers were counted from the highest dilution factors that produced a positive reading.

### Lung Viral Load

Lung viral load was determined at 72 h post-infection. Lungs of infected mice and control mice were homogenized in 1 mL of ice-cold PBS and frozen at −80°C for 14 h. Then, thawed samples were centrifuged at 2,000 × g for 10 min, and the supernatants were titrated by plaque forming assay as described above.

### Mouse Challenge Experiment

Female C57BL/6J WT mice were obtained from Vital River Laboratory Animal Center (Beijing, China). Type I interferon receptor (IFNAR1) knockout mice bred on a C57BL/6 background were kindly provided by Prof. Dan Portnoy (University of California, Berkeley, CA, USA). SOCS3-knockdown transgenic mice were generated as previously described (39). The transgenic mice were genotyped by PCR using specific primers shown in Figure S4A, and the transgenic founders were selected for further experiments. For infection, mice (5–6 weeks old, 17–19 g) were inoculated intranasally with 5 × 10^4^ PFU of IAV or 50 μL of sterile PBS. The daily weights of mice were monitored at indicated time points until 75% of initial weight. On the indicated time of post-infection (p.i.), mice were euthanized and their lungs were collected at the indicated time for further analysis by Western blotting, RT-PCR or RT-qPCR.

### Cell Stimulation and Enzyme-Linked Immunosorbent Assay

A549 cells were incubated with recombinant human IL-6 (50 ng/mL, PeproTech, Chicago, IL, USA) for 45 min, unless otherwise indicated. Cell culture supernatants from WSN-infected A549 cells (MOI = 1) were used as a source of virus-induced cytokines for stimulation of fresh cells. To quantify IL-6 protein production, culture supernatants from stimulated or infected cells as well as mouse lung tissue lysates were harvested and examined by enzyme-linked immunosorbent assay (ELISA) using the ready-SET-Go of human IL-6 analysis kit (eBioscience, CA, USA) or mouse IL-6 analysis kit (eBioscience, CA, USA) according to the manufacturer's instructions.

### Histopathological Analysis

C57BL/6 mice were intranasally inoculated with WSN virus for 3 days, and then sacrificed. Mouse lung tissues were collected and fixed in 4% paraformaldehyde and embedded with paraffin. Then, 4-mm-thick sections were prepared and stained with hematoxylin and eosin (H&E). The slides were visualized under an Olympus BH-2 microscope (Tokyo, Japan).

### Luciferase Assay

Luciferase assay was performed as previously described (36). Briefly, 293T cells in 24-well plates were co-transfected with 3 μg of plasmid expressing sh-SOCS3 (human) or plasmid expressing shRNA negative control (sh-control), 0.8 μg of pGL3-IL-6-promoter-Luc, and 30 ng of Renilla luciferase plasmid (pRL-TK, Promega, Madison, WI, USA) in serum-free medium for 4 h and then cultured in complete medium for another 6 h. Cells were infected with or without PR8 virus at MOI of 3 for 16 h. Luciferase activity driven by IL-6 promoter was measured using the dual-luciferase reporter assay system according to the manufacturer's instruction (Promega, Madison, WI, USA).

### Statistical Analysis

Data were presented as mean ± standard deviation (S.D.) from three independent experiments. Statistical analysis was performed by Student's *t-*test. *P* < 0.05 was considered to be significant.

### Accession Numbers

The data of the microarray have been deposited in NCBI's Gene Expression Omnibus under the accession number GSE80011.

## Results

### IAV Infection Induces Robust Expression of SOCS3 and IL-6 *in vitro* and *in vivo*

**Figure 1:**
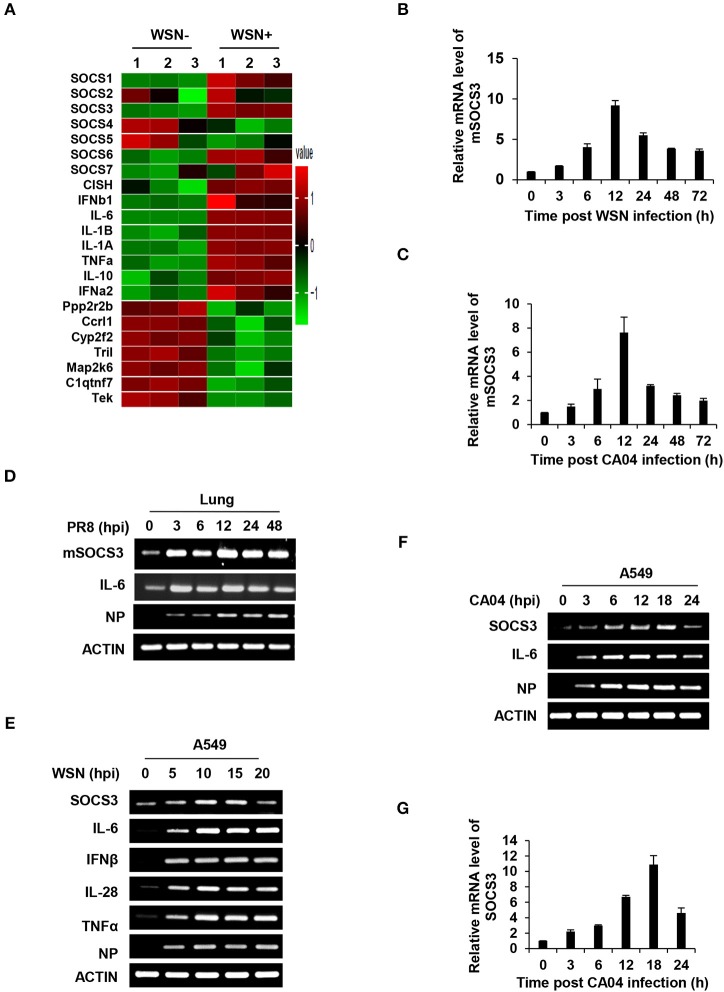
IAV infection induces robust expression of SOCS3 and IL-6 *in vitro* and *in vivo*. **(A)** The differentially expressed mRNAs (1399 upregulated and 693 downregulated) in lungs of C57BL/6 mice infected with A/WSN/33 influenza virus (5 × 10^4^ PFU) were detected by a cDNA microarray compared with uninfected controls (fold change >2, *p* < 0.05; GEO accession number GSE80011). The mRNA levels of SOCS members, IL-6 and representative differentially expressed genes are shown in the heatmap. **(B–D)** C57BL/6 mice were intranasally infected with WSN **(B)**, CA04 **(C)**, or PR8 **(D)** (5 × 10^4^ PFU) and were sacrificed at the indicated time. The mouse SOCS3 (mSOCS3) mRNA levels in the lungs were determined by quantitative RT-PCR (RT-qPCR) **(B,C)** or RT-PCR **(D)**. Shown are representative data from three independent experiments. The error bars represent the ±S.D. from the mean. **(E)** A549 cells were infected with WSN (MOI = 1) for indicated time, and the mRNA levels of SOCS3, IL-6, IFNβ, IL28, and TNFα were measured by RT-PCR. **(F,G)** A549 cells were infected with CA04 (MOI = 1) for indicated time, and the mRNA levels of SOCS3 were analyzed by RT-PCR **(F)** and RT-qPCR **(G)**. The average levels from three independent experiments are plotted. The error bars represent the ±S.D. from the mean.

Inflammatory response is an important component of the antiviral innate immunity. Disorders of inflammation deteriorate the health of patients and cause death in many viral diseases. To better understand *in vivo* regulation of inflammation during the IAV infection, cDNA microarrays were performed to investigate the genome-wide mRNA expression in C57BL/6 mice infected with or without WSN as previously described (40) (GEO accession number GSE80011). Among hundreds of differentially expressed genes, two important SOCS family members, SOCS1, and SOCS3 were significantly upregulated in the lung of infected mice (Figure 1A). This finding was consistent with the previous *in vitro* studies about increased SOCS expression in IAV-infected human bronchial epithelial cells (24). In addition, one of the most remarkable changes in the infected lung was greatly enhanced expression of a critical inflammatory cytokine IL-6 (Figure 1A). The data showed that IL-6 mRNA increased by more than 90-fold in the WSN-infected samples.

As SOCS3 acts as a potent suppressor of IL-6/STAT3 signaling, next, we focused on investigating the role of SOCS3 in IL-6-mediated inflammatory response triggered by IAV infection. Thus, the IAV-induced SOCS3 and IL-6 expression in mouse or human cells was further confirmed by RT-PCR and RT-qPCR. In line with the microarray data, a robust induction of SOCS3 and IL-6 was observed in the lungs of mice infected with several IAV H1N1 strains, including seasonal strain WSN, pandemic strain CA04, and lab adapted strain PR8 (Figures 1B–D and Figures S1A,B). Of note, the increase of SOCS3 in lungs of PR8-infected mice started at 3 h post-infection (hpi) in the early stage of IAV infection (Figure 1D). Moreover, the induced expression of SOCS3 and IL-6 was examined *in vitro* after the virus infection. As expected, significant upregulations of SOCS3 and IL-6 during IAV infection were found in IAV infected human A549 cells (Figures 1E–G), mouse macrophages Raw264.7 (Figure S1C), and human monocyte THP1 cells (Figure S1D). In addition, enhanced expression of IFNβ, IL28, antiviral proteins Mx1, and IFITM3 was also observed, indicating the effective IFN response to IAV infection in our system (Figures 1A,E and Figure S1E). It is noteworthy that the pandemic IAV strain CA04 induced remarkable early expression of SOCS3 in A549 cells (Figures 1F,G). Together, these results suggest a crucial role of SOCS3 in the interaction between the virus and host.

### Expression of SOCS3 at Early Stage of IAV Infection Is Independent of IL-6 and Type I IFNs

**Figure 2:**
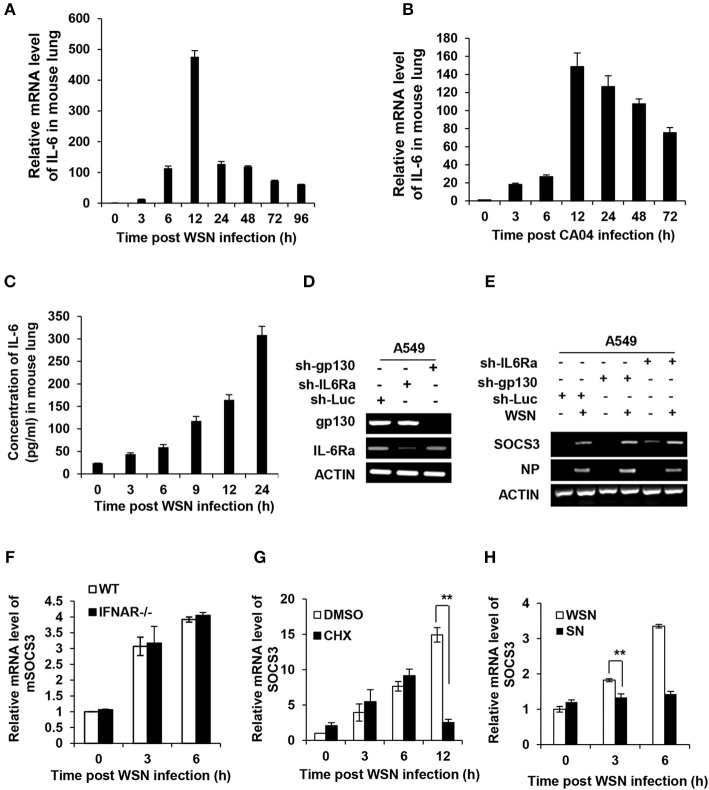
Expression of SOCS3 at early stage of IAV infection is independent of induction of cytokines including IL-6. **(A,B)** C57BL/6 mice were intranasally infected with WSN **(A)** or CA04 **(B)** (5 × 10^4^ PFU) for the indicated time and then sacrificed. The mRNA levels of IL-6 in the lungs were examined via RT-qPCR. **(C)** C57BL/6 mice were intranasally infected with WSN **(A)** or CA04 **(B)** (5 × 10^4^ PFU) for the indicated time and then sacrificed. The protein levels of IL-6 were measured via ELISA. Shown are representative data from three independent experiments. The error bars represent the ±S.D. from the mean. **(D)** shRNA based-knockdown efficiency of gp130 and IL-6 receptor α (IL-6Ra) were determined by RT-PCR. **(E)** A549 cells expressing shRNAs targeting gp130, IL-6Rα, or luciferase (Luc) were infected with or without WSN for 3 h, and then the expression of SOCS3 was examined by RT-PCR. Shown are representative blots from three independent experiments. **(F)** The IFNAR1 knockout (IFNAR^−/−^) or WT mice were infected with WSN virus for indicated time, and then the mSOCS3 levels in lungs were determined by RT-qPCR. **(G)** A549 cells were treated with DMSO (0.1%) or CHX (1 ng/mL) for 30 min, and then infected with WSN (MOI = 1) for indicated time. The expression of mSOCS3 was determined by RT-qPCR. **(H)** A549 cells were infected by WSN (MOI = 1) for indicated time. Supernatants (SN) derived from these cell cultures were used to stimulate the native A549 for 1 h. Both infected cells and SN-stimulated cells were used to analyze mSOCS3 expression via RT-qPCR. The average levels from three independent experiments are plotted. The error bars represent the ±S.D. from the mean, ^**^P < 0.01.

The SOCS3 expression is known to be stimulated by IL-6, IFNs and other cytokines (25, 32, 41, 42). To unveil how SOCS3 expression is upregulated at the early stage of IAV infection, we determined whether its transcription triggered by IAV was dependent on cytokines. As shown in the microarray data described above, IL-6 mRNA increased by more than 90-fold in lungs of the WSN-infected mice (Figure 1B). Interestingly, results obtained from analysis by RT-PCR, RT-qPCR, and ELISA showed that IL-6 mRNA and protein levels were substantially upregulated in the PR8, WSN, or CA04 infected mice or human A549 cells (Figures 1E–G, 2A–C), and IAV-induced SOCS3 appeared to occur concomitantly with the expression of IL-6 (Figures 1C–G). These observations prompted us to examine whether SOCS3 was induced due to IL-6-mediated signaling activated during the early infection. Thus, we generated A549 cell lines stably expressing shRNAs specifically targeting IL-6 receptor subunits glycoprotein 130 (gp130) or IL-6Ra and found that knockdown of IL-6 receptor subunits did not affect the mRNA levels of SOCS3 at early stage of the infection (Figures 2D,E).

Next, we employed IFNAR1 knockout mice to test whether type I IFNs were involved in early induction of SOCS3 by IAV. We observed that the induction of SOCS3 expression at early stages (3 and 6 hpi) was not impaired by the knockout of IFNAR1 (Figure 2F and Figure S2A). Furthermore, we asked whether the newly produced other cytokines were responsible for the early induction of SOCS3 by IAV. To address this possibility, cycloheximide (CHX), a translation inhibitor, was used to block the *de novo* synthesis of proteins, including all cytokines, before and during viral infection. Although protein synthesis including viral NP protein was dramatically inhibited upon CHX treatment, SOCS3 mRNA levels at early stages (3–6 hpi) were not significantly reduced (Figure 2G and Figure S2B). However, markedly reduced induction of SOCS3 was shown at late stage of the infection (12 hpi), implying that *de novo* synthesized proteins were required for efficient SOCS3 expression at late stage but not for early stage of IAV infection (Figure 2G and Figure S2B). To further confirm this finding, we treated A549 cells with cell culture supernatants derived from cells infected with the virus at the early stage. We found that these supernatants failed to stimulate the SOCS3 expression, as compared with viral infection control (3 hpi) (Figure 2H and Figure S2C). Taken together, these data indicate that IAV itself, but not IAV-induced cytokines including IL-6 or newly synthesized viral proteins, might trigger SOCS3 expression at the early infection stage.

### IAV Impairs IL-6-stimulated STAT3 Activation by Upregulation of SOCS3

**Figure 3:**
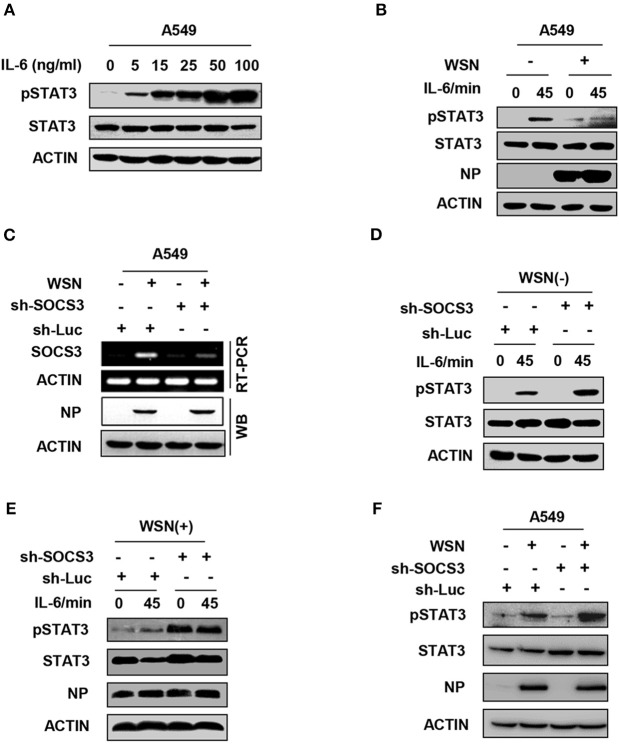
IAV impairs IL-6-stimulated STAT3 activation by upregulation of SOCS3. **(A)** A549 cells were stimulated with recombinant human IL-6 protein at the indicated concentration, and the phosphorylation of STAT3 was examined by Western blotting. Shown are representative data from three independent experiments. **(B)** A549 cells infected with WSN (MOI = 1) for 15 h (WSN+) or not infected (WSN-) were stimulated with human IL-6 (50 ng/mL) for indicated time. Cell lysates were used to analyze the phosphorylation of STAT3 by Western blotting. **(C)** A549 cells expressing shRNAs targeting either SOCS3 or control luciferase (Luc) were infected with WSN (MOI = 1) for 15 h. RT-PCR was performed to examine the interference efficiency. Shown are representative data from three independent experiments. **(D,E)** A549 cells expressing sh-Luc or sh-SOCS3 were infected without **(D)** or with **(E)** WSN virus and then treated with IL-6 (50 ng/mL) for indicated times. pSTAT3 levels of the cell lysates were examined by Western blotting. **(F)** A549 cells expressing shRNAs targeting SOCS3 or luciferase were infected with WSN for 15 h. Cell lysates were analyzed by Western blotting with indicated antibodies. Shown are representative blots from three independent experiments.

Among cytokines, IL-6 is the major mediator of STAT3 activation. IL-6 primarily activates the JAK2-STAT3 signaling pathway through binding to its receptor complex gp130/IL-6R on the host cell surface, and then initiates the transcription of inflammatory genes. SOCS3 functions as a key inhibitor of JAK2-STAT3 signaling (43, 44). We hypothesized that to facilitate its replication, IAV might suppress the IL-6-induced activation of STAT3 through enhancing the expression of SOCS3. To directly determine whether IL-6-activated STAT3 was inhibited in IAV-infected host, we used purified IL-6 protein to stimulate cells. As expected, IL-6 was able to activate the JAK-STAT3 signal pathway in A549 cells (Figure 3A). Strikingly, the level of IL-6-induced STAT3 phosphorylation was reduced in IAV-infected cells as compared with that in control cells (Figure 3B). Next, we further investigated involvement of IAV-induced SOCS3 in inhibition of STAT3 phosphorylation during IAV infection. To this end, we generated a SOCS3-knockdown A549 cell line using SOCS3-specific shRNA (Figure 3C). Indeed, we observed that the impairment of IL-6-stimulated STAT3 activation by IAV was abrogated in SOCS3-knockdown cells (Figures 3D,E). Furthermore, silencing SOCS3 expression significantly enhanced the phosphorylation of STAT3 in WSN-infected cells (Figure 3F and Figure S3). These data indicate that IAV-induced SOCS3 impairs IL-6-stimulated STAT3 phosphorylation during viral infection.

### Targeted Disruption of SOCS3 Expression Decreases Viral Replication During IAV Infection *in vitro* and *in vivo*

**Figure 4:**
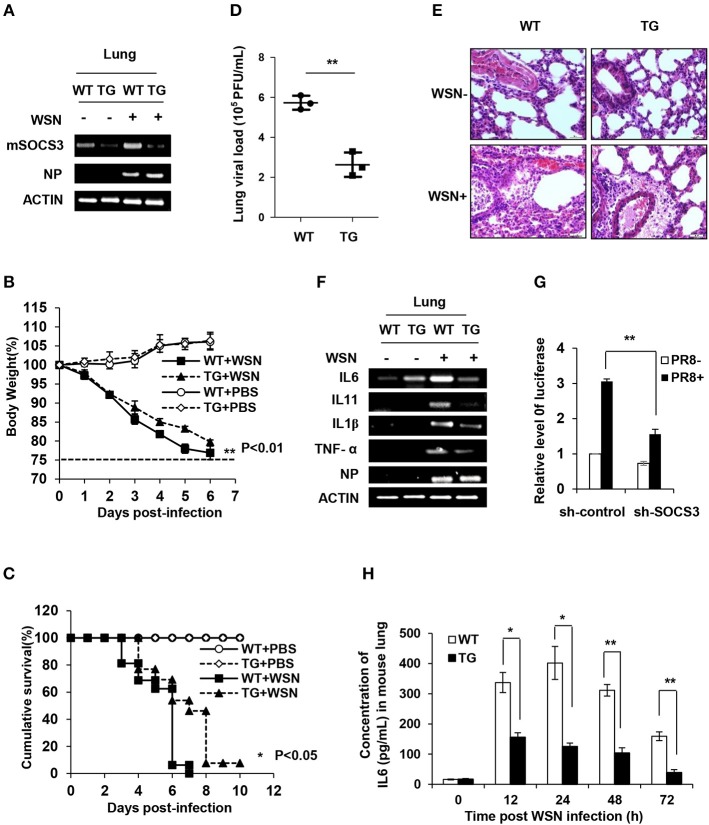
Targeted disruption of SOCS3 expression decreases viral replication during IAV infection *in vitro* and *in vivo*. **(A)** The mRNA levels of mSOCS3 in lung tissues from SOCS3-knockdown TG mice and WT littermates were examined via RT-PCR after WSN infection for 24 h. **(B)** Shown is the body percentage weight change of WT (*n* = 13) and TG (*n* = 13) mice intranasally infected with WSN or PBS. Body weight was measured daily. Data are shown as mean ± S.D. from three independent experiments. ^**^P < 0.01 (Student's *T*-test). The dashed line indicates 75% of initial body weight. **(C)** Survival rates of WT mice (*n* = 13) and TG mice (*n* = 13) intranasally infected with or without WSN are shown. Mice were monitored for 10 days or sacrificed when showing hind limb paralysis or weight loss over 25%. ^*^P < 0.05 (Gehan-Breslow-Wilcoxon test). **(D)** Shown is the viral load in the lungs of WT and TG mice infected with WSN for 3 days. **(E)** Shown are the representative lung photomicrographs (magnification, ×400) of WT and TG mice infected with or without WSN (3 dpi) stained with hematoxylin and eosin (HE). The pathological changes of lungs in WT mice were severer than those in TG mice. Scale bars, 30 μm. **(F)** TG mice and WT mice were intranasally infected with WSN for 24 h. The expression of indicated genes in mouse lungs was examined by RT-PCR. **(G)** 293T cells were co-transfected with pGL6-IL-6-promoter-luc and plasmid expressing sh-SOCS3 or control shRNA (sh-control), and then infected with PR8 virus (MOI = 3) for 16 h. The relative activity of luciferase driven by IL-6 promoter in cell lysates was normalized to renilla luciferase activity and displayed as mean ± S.D. from three independent experiments. **(H)** TG mice and WT mice were intranasally infected with WSN for indicated time points. The expression of IL-6 in the lungs was examined by ELISA. Shown are representative data from three independent experiments. Data are shown as mean ± S.D.; *n* = 3; ^*^P < 0.05; ^**^P < 0.01 (Student's *T*-test).

To determine the functional involvement of SOCS3 in antiviral response *in vivo*, we utilized SOCS3-knockdown transgenic (TG) mice as previously described (39) (Figure 4A and Figure S4A). The TG mice and wild-type (WT) littermates were then challenged by WSN virus intranasally. Surprisingly, silence of SOCS3 provided significant protection of TG mice against IAV infection (Figures 4B–D). The TG mice exhibited notably lower weight loss and higher survival rate in comparison with WT littermates after IAV infection (Figures 4B,C). The results showed that the body weight of WT and TG mice dropped ~22 and 16.7% at 5 days post-infection (dpi), respectively (Figure 4B). At 6 dpi, about 90% of WT mice succumbed to IAV infection, whereas ~50% of SOCS3-knockdown TG mice still survived under the same condition (Figure 4C). Importantly, we found that the virus load in the lungs of TG mice at 3 dpi was significantly lower than that of WT mice (Figure 4D). Consistently, the suppression of IAV replication was also observed *in vitro* from the A549 cells expressing SOCS3 shRNA (Figure S4B). Furthermore, the lungs of infected TG mice displayed less necrosis and less filtration or damage as compared to WT controls (Figure 4E and Figure S4C). These data suggest that SOCS3, as an important negative regulator of IL-6-JAK-STAT3 pathway, might be hijacked by IAV to overcome the effects of IL-6 activated STAT3 signaling. Thus, IAV-induced high level of SOCS3 is in favor of viral replication and thereby promotes influenza pathogenesis.

Aberrant innate response, with excessive IL-6 production and robust recruitment of inflammatory leukocytes to the lung, was believed to critically contribute to the disease progression and the ultimate death of humans or animals infected by highly virulent influenza virus. However, the mechanism underlying the excessive expression of IL-6 during IAV infection remains largely unclear. Since SOCS3 is a key regulator of IL-6/STAT3 signaling and silencing SOCS3 expression in genetically modified mice can effectively protect the animals against IAV infection, we hypothesized that altered SOCS3 expression may affect IL-6 production and thus IAV pathogenesis. To test this possibility, expression of IL-6 was examined in SOCS3-knockdown A549 cells infected with or without IAV (Figure S4D). Indeed, silencing SOCS3 resulted in a significant decrease in expression of inflammatory cytokines IL-6, IL-1β, and TNFα, but little change in the levels of type I and III IFNs after IAV infection (Figure S4D). Consistently, the results from the lungs of IAV-infected TG and WT mice showed that knockdown of SOCS3 caused a decreased expression of IL-6, IL-1β, IL-11, and TNFα (Figure 4F). The data suggest that SOCS3 might be involved in the regulation of a common pathway that drives the transcription of these inflammatory genes. Moreover, IL-6 promoter-driven luciferase reporter gene assay consistently showed a significant decrease in IL-6 promoter activity in cells expressing SOCS3 shRNA, confirming that SOCS3 deficiency is critically associated with the downregulation of IL-6 expression (Figure 4G).

Next, we determined the regulatory role of SOCS3 in the activation of STAT3 and in the expression of the inflammatory cytokines during the IAV infection *in vivo*. To this end, SOCS3-knockdown TG mice were infected with IAV for different times. As expected, silencing SOCS3 markedly enhanced activation of STAT3 (Figure S4E). Furthermore, IL-6 mRNA and protein levels were determined by RT-PCR, real-time PCR and ELISA, respectively. Similarly, significant reduction in IL-6 mRNA and protein was shown in the lung of TG mice as compared with that of WT control during IAV infection (Figure 4H and Figures S4F,G). Taken together, these results reveal that SOCS3 might play a crucial role not only in suppressing IL-6/STAT3 signaling but also in feedback regulation of IL-6 expression during the viral infection. Thus, the antiviral effect derived from SOCS3 silencing is likely due to the reduced production of inflammatory cytokines and the sustained activation of STAT3, which may establish a favorable microenvironment for innate immunity against IAV infection.

### Altering STAT3 Activity Has a Significant Effect on IL-6 Expression Induced by IAV Infection

**Figure 5:**
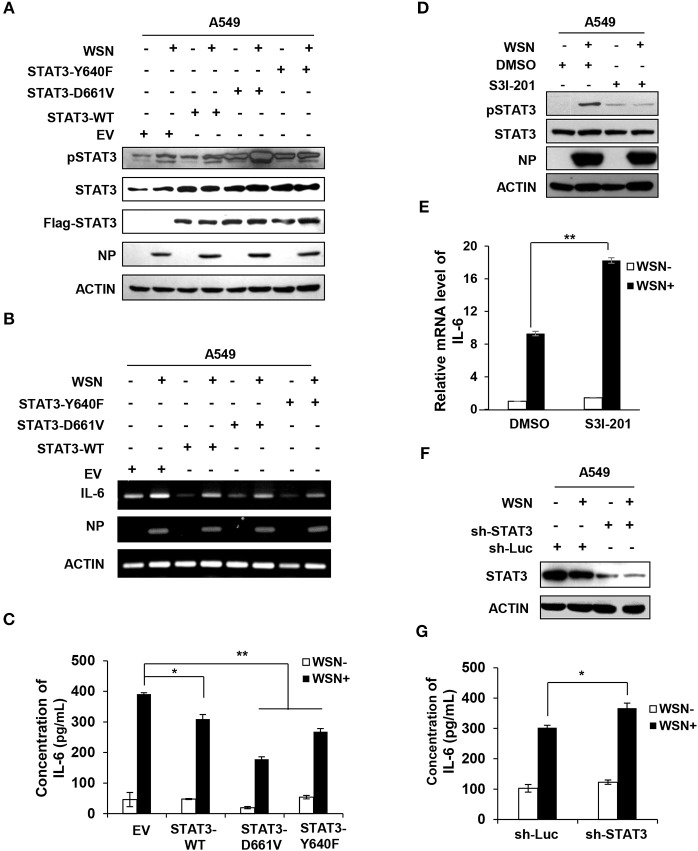
Altering STAT3 activity has significant effect on IL-6 expression. **(A–C)** A549 cells stably expressing STAT3-WT, STAT3-D661V, STAT3-Y640F, and EV control were infected with or without WSN (MOI = 0.5) for 15 h. The phosphorylation of STAT3 in these cells was detected by Western blotting **(A)**, and the mRNA or protein levels of IL-6 in these cells were examined by RT-PCR **(B)** or ELISA **(C)**. The error bars represent the ±S.D. from the mean,^*^P < 0.05; ^**^P < 0.01. **(D–E)** A549 cells were pretreated with DMSO (0.1 %) or S3I-201 (20 μM) for 2 h, and then infected with or without WSN (MOI = 0.5) for 15 h. The phosphorylation of STAT3 in these cells was detected by Western blotting, and shown are representative blots from three independent experiments (D). The mRNA levels of IL-6 were determined by RT-qPCR **(E)**. Plotted are the average levels from three independent experiments. The error bars represent the ±S.D. from the mean, ^**^P < 0.01. **(F,G)** A549 cells expressing shRNAs targeting either STAT3 or control luciferase (Luc) were infected with or without WSN (MOI = 1) for 15 h. Cell lysates were analyzed by Western blotting with STAT3 antibodies **(F)**, and IL-6 protein levels in these samples were determined by ELISA **(G)**. Data are shown as mean ± S.D.; *n* = 3; ^*^P < 0.05 (Student's *T*-test).

Since our results presented above exhibited that IL-6-activated STAT3 was inhibited by IAV-induced SOCS3 and knockdown of SOCS3 downregulated IL-6 during the viral infection, we asked whether elevated activity of STAT3 caused by silencing SOCS3 affected IL-6 expression. To address this possibility, we examined the effect of forced STAT3 activation on IL-6 production. Thus, we generated A549 cell lines stably expressing either empty vector control, wild-type STAT3, or two constitutively active mutants of STAT3 (Y640F and D661V). The enhanced phosphorylation of active forms of STAT3 was verified by Western blotting (Figure S5A). Moreover, constitutive activation of these STAT3 proteins was further confirmed in IAV infected cells (Figure 5A). Of interest, overexpression of WT or constitutively active STAT3 markedly reduced mRNA expression of IL-6 (Figure 5B). Similar results were obtained from experiments measuring the IL-6 protein levels in these cells (Figure 5C). The observations are consistent with studies that both mRNA and protein levels of IL-6 enhanced in SOCS3-knockdown cells (45, 46), and suggest that persistent activation of STAT3 may be responsible for the reduced expression of IL-6. Consistently, we observed that inactivation of STAT3 by inhibitor S3I-201 (Figure 5D), or knockdown of STAT3 by shRNA caused a significant increase in IL-6 mRNA levels in WSN infected A549 cells (Figures 5E,F and Figure S5B). Similar results were gained from ELISA measuring the IL-6 protein levels in the WSN-infected STAT3-knockdown cells (Figure 5G). Therefore, these observations imply that suppression of cytokine signaling by IAV-induced SOCS3 may contribute to excessive production of IL-6 in the virus infected host.

### SOCS3 and Activated STAT3 Are Involved in Regulating the Function of NFκB

**Figure 6:**
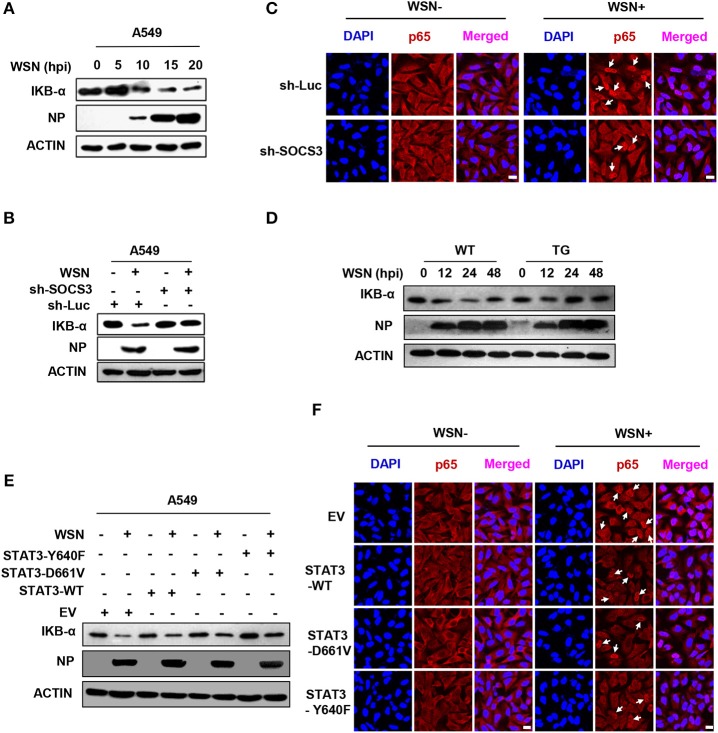
SOCS3 and activated STAT3 are involved in regulating the function of NFκB. **(A)** IKB-α levels of A549 cells infected with WSN for indicated times were detected by Western blotting. **(B)** A549 cells expressing sh-SOCS3 or sh-Luc were infected with or without WSN for 15 h. IKB-α levels were examined by Western blotting. Shown are representative blots from three independent experiments. **(C)** A549 cells stably expressing sh-SOCS3 or control were infected with or without WSN for 15 h. Immunofluorescence staining was performed using an anti-NFκB p65 antibody to detect translocation of NFκB (red). The nuclei were stained with DAPI (blue). **(D)** The expression of IKB-α in lung tissues from SOCS3-knockdown TG mice and WT littermates were examined via Western blotting after WSN (5 × 104 PFU) infection at the indicated times. Shown are representative data from three independent experiments. **(E,F)** A549 cells stably expressing STAT3-D661V, STAT3-Y640F, STAT3-WT or EV control were infected with or without WSN for 15 h. IKB-α levels in these cells were determined by Western blotting (E). Immunofluorescence staining was performed to detect NFκB p65 (red) in cells infected with or without WSN (F). The nuclei were stained with DAPI (blue). Shown are representative results from three independent experiments. Scale bars, 10 μm.

NFκB is an important transcription factor to drive the expression of many immune-related genes. It is also well-known that NFκB is a major transcription factor governing the transcription of IL-6 (12). IAV infection induced NFκB activation, as an obvious decrease in the level of its inhibitor IKBα was detected (Figure 6A). To determine whether silencing SOCS3 expression affected IL-6 production through modulating the function of NFκB, we investigated the effect of SOCS3 disruption on NFκB activity *in vitro* and *in vivo*. Indeed, both Western blotting and immunofluorescence staining showed increased IKBα and decreased nuclear p65 form of NFκB in IAV-infected SOCS3-knockdown cell and lung of TG mice as compared to WT host, suggesting that silencing SOCS3 may result in a suppression of NFκB upon viral infection (Figures 6B–D). Furthermore, we explored whether the phosphorylated STAT3 is involved in regulating NFκB activation. To this end, IKBα and nuclear p65 levels were examined in cells expressing either empty vector, WT or forced activated STAT3. We observed that an elevation of IKBα level and a reduction in nuclear p65 were observed in IAV-infected cells overexpressing WT or constitutively active STAT3, implying that persistent activation of STAT3 may impair NFκB activity (Figures 6E,F). The results are in accordance with a previous report that NFκB target gene iNOS was suppressed by STAT3 via a direct interaction between STAT3 and p65 (47). Taken together, our findings suggest that IAV-induced SOCS3 is associated with the upregulation of IL-6 expression likely through activating NFκB. One mechanism for the enhanced NFκB activation in the presence of high levels of SOCS3 might operate through suppressing STAT3 activity.

## Discussion

The innate immune system is the first line of defense against IAV infection by inducing expression of cytokines and chemokines. However, excessive production of these molecules named as “cytokine storm,” especially in the case of infections with highly pathogenic influenza strains, contributes to disease severity. For example, severe seasonal influenza in ferrets correlates with increased IL-6 induction (48). However, the mechanism underlying aberrant expression and regulation of IL-6 during the viral infection is still poorly understood (49). In this study, we found that IAV infection induced a dramatic upregulation of IL-6, which was likely through enhanced activation of the transcription factor NFκB. In general, the fluctuation of IL-6 is important for the pro-inflammatory function to modulate and switch the innate immune response to adaptive immune response (19, 50). For instance, IL-6 secreted by the local infected cells recruits neutrophils at the early stage of infection, and then IL-6 secreted by neutrophils attracts macrophages and monocytes to the inflamed sites 24 h after the initial infection (6, 19). Besides, blood-borne IL-6 promotes the differentiation of effector T cells, memory T cells and antibody-producing B cells (19, 50, 51). As a feedback, the negative regulator SOCS3 is expressed, which is driven by IL-6-activated transcription factor STAT3. The feedback regulation of IL-6-activated STAT3 signaling by SOCS3 is critically required for the tight control of inflammation (34, 44).

SOCS3 not only is induced by IL-6/STAT3 signaling, but also like IL-6, is an NFκB target gene. Therefore, IAV-induced robust expression of SOCS3 might be a combined result from at least two or more sets of regulations (24, 25). Previous evidence suggests that an abnormally high level of IL-6 can cause severe tissue damages and increase disease severity (50). However, the exact relationship between IL-6 and SOCS3, especially the induction and the functional involvement of SOCS3 in regulating IL-6 production during the viral infection, remains unclear. In this study, we noticed that the early induction of SOCS3 in IAV-infected cells was independent of IL-6, implying that IAV may have evolved to establish a repressive circumstance for IL-6/STAT3 signaling, which is beneficial for the initiation of viral replication. We assumed that the suppression of IL-6/STAT3 signaling by the early production of SOCS3 might lead to elevation of IL-6 in late stage of IAV infection. Indeed, the involvement of SOCS3 in the regulation of IL-6 expression was demonstrated by our *in vitro* and *in vivo* experiments showing that depletion of SOCS3 significantly reduced the viral infection-triggered expression of IL-6. These findings suggest that impaired IL-6/STAT3 signaling due to inhibitory effect by high levels of SOCS3 contributes to excessive production of IL-6 during IAV infection.

We further discovered that the low expression of IL-6 caused by the deficiency of SOCS3 could effectively inhibit the IAV replication *in vivo* and *in vitro*. Our *in vivo* anti-IAV effect of SOCS3 deficiency is in accordance with a previous study which showed that expression of SOCS3 was downregulated by injected recombinant IL-7 in mice infected with lymphocytic chorimeningitis virus (LCMV) variant clone 13, and downregulation of SOCS3 enhanced cytokine signaling and thereby the LCMV was efficiently eliminated (9). Similar results have been also found in patients with chronic hepatitis C viral genotype 1, in whom the overexpression of SOCS3 in liver tissue is associated with a poorer treatment outcome (29), as well as in IAV-infected SOCS3-knockout mouse embryonic fibroblasts (MEFs), in which progeny virus titers are significantly reduced compared to control (25). Recently, another IAV-induced SOCS family member SOCS1 has been found to consistently impair viral clearance and exacerbate lung injury during IAV infection (52). Furthermore, the antiviral function of IL-7 is related to the activated IL-6/STAT3 signaling and increased amount of active T cells (9). In this study, we speculate that enhanced STAT3 activity and relatively low level of IL-6 might be important signals to recruit an appropriate amount of immune cells, which not only can clear the virus effectively but also avoid serious lung damage caused by the excessive IL-6 storm. As such, the IAV-induced robust expression of SOCS3 causes a feedback increase in IL-6 expression, impairs viral clearance and promotes the lung injury *in vivo*. However, the precise mechanism by which the deficiency of SOCS3, enhanced activation of STAT3 and decreased expression of IL-6 influence host antiviral immunity remains unclear. Although the absence of SOCS3 has been shown to increase the IL-6-induced IFN-like program and to sustain the phosphorylation of STAT1 in the IFNs response (20, 25, 27, 28, 53), little is known about the roles of phosphorylated STAT3 and SOCS3 in regulating antiviral ISGs expression. It has been shown that IL-6-activated STAT3 induces significant upregulation of IFITM3 in T cells (7). IFITM3 is a critical antiviral protein in innate immune response to prevent the viral cytosolic entry from the endosome (54–56). Therefore, a possible mechanism underlying the effect of silencing SOCS3 on antiviral response might be through the STAT3-mediated expression of IFITM3 or other ISGs in cells. Further investigation is needed to address this issue.

In addition, an interesting finding here is that the loss of SOCS3 strongly reduced NFκB activity and IL-6 levels *in vitro* and *in vivo*. Our results are, to some extent, in contrast to a previous report that overexpression of SOCS3 reduces NFκB activity (24). Possibly, a different MOI of A/Scotland/20/74 (H3N2) virus and a different cell line for the infection used in the other research (24) might cause the discrepancy, since the antiviral responses are associated with viral titers and host cell type specific. However, our results are consistent with other studies that forced expression of SOCS3 can activate RelA-dependent NFκB and transcription of IL-6 in macrophages and muscle cells (57–59). In this study, we found that SOCS3 deficiency inhibited the replication of IAV. Likely, the low viral propagation in SOCS3 knockdown cells is a feedback signal to reduce the high activity of NFκB and thereby impair the transcription of IL-6 to appropriate levels to avoid inflammation-associated damages. Nevertheless, the mechanism underlying this process is still unknown, and more efforts are required to tackle this question. On the other hand, a possible mechanism for the reduced IL-6 expression in SOCS3-knockdown host may be associated with the elevated pSTAT3 that crosstalks and inhibits the transcriptional activity of NFκB, and thereby suppresses transcription of IL-6, whose promoter contains both NFκB and STAT3 binding sites (60, 61). It has been observed that pancreatitis-associated protein I (PAP I), an inducible anti-inflammatory protein, blocks NFκB through activating phosphorylation and nuclear translocation of STAT3 in pancreatic acinar cells, resulting in decreased expression of IL-6 and TNFα (62). Notably, several experiments have demonstrated the crosstalk between NFκB and STAT3 in the nucleus, including direct interaction between NFκB-p65 and pSTAT3, and p300-involved cooperation or competition at promoters/enhancers of target genes (47, 60, 61, 63, 64). However, in human melanoma cells where STAT3 is persistently activated, the STAT3-NFκB-p300 protein complex can cooperatively activate the expression of many oncogenic/inflammatory genes (61). We assume that the different microenvironments in the various tissues might initiate distinct unknown mechanisms of STAT3-NFκB crosstalk. This will be explored in the future.

In conclusion, here we provide novel evidence that IAV triggered robust expression of SOCS3, a negative regulator of IL-6-STAT3 signaling, in an IL-6-independent manner at the early stage of the virus infection. This was beneficial for IAV infection and propagation by control of IL-6-STAT3 signaling. However, disruption of IL-6-STAT3 signaling by IAV-induced SOCS3 results in an adaptive increase in IL-6 expression by host in response to the viral infection, which may lead to excessive production of IL-6 and promote lung damage. Our findings establish a critical role for the IL-6-STAT3-SOCS3 axis in the pathogenesis of IAV and shed light on the complicated strategies taken by IAV to antagonize the host innate immunity.

## Data Availability

The datasets for this manuscript are not publicly available because they are currently private (GEO accession number: GSE80011) and scheduled to be released on Apr 30, 2021. Requests to access the datasets should be directed to J-LC.

## Ethics Statement

Mice were bred and housed in colony room at Institute of Microbiology, Chinese Academy of Sciences. The humidity (50–70%) and temperature (21–24°C) were controlled. The room was maintained on a 12:12 light: dark cycle and water was available. The mice lived in autoclaved individual ventilated cages (IVC) in group up to five same-sex each cage. The animal experimental protocol used in this study were in accordance with the guidelines contained in the International Guiding Principles for Biomedical Research Involving Animals (CIOMS & ICLAS, 2019) and were approved by the Research Ethics Committee of Institute of Microbiology, Chinese Academy of Sciences (permit number APIMCAS2017045). All mouse experimental procedures were performed in accordance with the Regulations for the Administration of Affairs Concerning Experimental Animals approved by the State Council of People's Republic of China.

## Author Contributions

SL, RY, and J-LC designed the study, analyzed the data, and wrote the manuscript. SL, RY, BC, QP, YC, and JH performed the experiments. LZ contributed to generation of SOCS3-knockdown transgenic mice. WL and J-LC analyzed the data and revised the manuscript. SW contributed to critical comments and revision of the manuscript. All authors meet the criteria for authorship.

### Conflict of Interest Statement

The authors declare that the research was conducted in the absence of any commercial or financial relationships that could be construed as a potential conflict of interest.

## References

[1] Zhu G Xu Y Cen X Nandakumar KS Liu S Cheng K. Targeting pattern-recognition receptors to discover new small molecule immune modulators. Eur J Med Chem. (2018) 144:82–92. 10.1016/j.ejmech.2017.12.026 29268133

[2] Reikine S Nguyen JB Modis Y. Pattern recognition and signaling mechanisms of RIG-I and MDA5. Front Immunol. (2014) 5:342. 10.3389/fimmu.2014.00342 25101084 PMC4107945

[3] Iwasaki A Pillai PS. Innate immunity to influenza virus infection. Nat Rev Immunol. (2014) 14:315–28. 10.1038/nri3665 24762827 PMC4104278

[4] Ramos I Fernandez-Sesma A. Modulating the innate immune response to influenza A virus: potential therapeutic use of anti-inflammatory drugs. Front Immunol. (2015) 6:361. 10.3389/fimmu.2015.00361 26257731 PMC4507467

[5] Rose-John S. Interleukin-6 family cytokines. Cold Spring Harb Perspect Biol. (2018) 10:a028415. 10.1101/cshperspect.a028415 28620096 PMC5793756

[6] Dienz O Rud JG Eaton SM Lanthier PA Burg E Drew A. Essential role of IL-6 in protection against H1N1 influenza virus by promoting neutrophil survival in the lung. Mucosal Immunol. (2012) 5:258–66. 10.1038/mi.2012.2 22294047 PMC3328598

[7] Dienz O Eaton SM Bond JP Neveu W Moquin D Noubade R. The induction of antibody production by IL-6 is indirectly mediated by IL-21 produced by CD4+ T cells. J Exp Med. (2009) 206:69–78. 10.1084/jem.20081571 19139170 PMC2626667

[8] Short KR Kroeze EJ Fouchier RA Kuiken T. Pathogenesis of influenza-induced acute respiratory distress syndrome. Lancet Infect Dis. (2014) 14:57–69. 10.1016/S1473-3099(13)70286-X 24239327

[9] Pellegrini M Calzascia T Toe JG Preston SP Lin AE Elford AR. IL-7 engages multiple mechanisms to overcome chronic viral infection and limit organ pathology. Cell. (2011) 144:601–13. 10.1016/j.cell.2011.01.011 21295337

[10] Parish IA Kaech SM. IL-7 knocks the socs off chronic viral infection. Cell. (2011) 144:467–8. 10.1016/j.cell.2011.01.038 21335230 PMC4380280

[11] Yu X Zhang X Zhao B Wang J Zhu Z Teng Z. Intensive cytokine induction in pandemic H1N1 influenza virus infection accompanied by robust production of IL-10 and IL-6. PLoS ONE. (2011) 6:e28680. 10.1371/journal.pone.0028680 22174866 PMC3235144

[12] Tanaka T Narazaki M Kishimoto T. IL-6 in inflammation, immunity, and disease. Cold Spring Harb Perspect Biol. (2014) 6:a016295. 10.1101/cshperspect.a016295 25190079 PMC4176007

[13] Hillmer EJ Zhang H Li HS Watowich SS. STAT3 signaling in immunity. Cytokine Growth Factor Rev. (2016) 31:1–15. 10.1016/j.cytogfr.2016.05.001 27185365 PMC5050093

[14] Cui W Liu Y Weinstein JS Craft J Kaech SM. An interleukin-21-interleukin-10-STAT3 pathway is critical for functional maturation of memory CD8+ T cells. Immunity. (2011) 35:792–805. 10.1016/j.immuni.2011.09.017 22118527 PMC3431922

[15] Tumang JR Holodick NE Vizconde TC Kaku H Frances R Rothstein TL. A CD25(-) positive population of activated B1 cells expresses LIFR and responds to LIF. Front Immunol. (2011) 2:6. 10.3389/fimmu.2011.00006 22566797 PMC3342026

[16] Cho JJ Xu Z Parthasarathy U Drashansky TT Helm EY Zuniga AN. Hectd3 promotes pathogenic Th17 lineage through Stat3 activation and Malt1 signaling in neuroinflammation. Nat Commun. (2019) 10:701. 10.1038/s41467-019-08605-3 30741923 PMC6370850

[17] Deenick EK Pelham SJ Kane A Ma CS. Signal transducer and activator of transcription 3 control of human T and B cell responses. Front Immunol. (2018) 9:168. 10.3389/fimmu.2018.00168 29472924 PMC5810249

[18] Knosp CA Johnston JA. Regulation of CD4+ T-cell polarization by suppressor of cytokine signalling proteins. Immunology. (2012) 135:101–11. 10.1111/j.1365-2567.2011.03520.x 22044277 PMC3277712

[19] Scheller J Chalaris A Schmidt-Arras D Rose-John S. The pro- and anti-inflammatory properties of the cytokine interleukin-6. Biochim Biophys Acta. (2011) 1813:878–88. 10.1016/j.bbamcr.2011.01.034 21296109

[20] Johnston JA O'Shea JJ. Matching SOCS with function. Nat Immunol. (2003) 4:507–9. 10.1038/ni0603-507 12774070

[21] Akhtar LN Benveniste EN. Viral exploitation of host SOCS protein functions. J Virol. (2011) 85:1912–21. 10.1128/JVI.01857-10 21084484 PMC3067810

[22] Yasukawa H Ohishi M Mori H Murakami M Chinen T Aki D Yoshimura, IL-6 induces an anti-inflammatory response in the absence of SOCS3 in macrophages. Nature Immunol. (2003) 4:551–6. 10.1038/ni938 12754507

[23] Delgado-Ortega M Marc D Dupont J Trapp S Berri M Meurens F. SOCS proteins in infectious diseases of mammals. Vet Immunol Immunopathol. (2013) 151:1–19. 10.1016/j.vetimm.2012.11.008 23219158 PMC7112700

[24] Pothlichet J Chignard M Si-Tahar M. Cutting edge: innate immune response triggered by influenza A virus is negatively regulated by SOCS1 and SOCS3 through a RIG-I/IFNAR1-dependent pathway. J Immunol. (2008) 180:2034–8. 10.4049/jimmunol.180.4.2034 18250407

[25] Pauli EK Schmolke M Wolff T Viemann D Roth J Bode JG. Influenza A virus inhibits type I IFN signaling via NFkappaB-dependent induction of SOCS-3 expression. PLoS Pathog. (2008) 4:e1000196. 10.1371/journal.ppat.1000196 18989459 PMC2572141

[26] Okabayashi T Kariwa H Yokota S Iki S Indoh T Yokosawa N. Cytokine regulation in SARS coronavirus infection compared to other respiratory virus infections. J Med Virol. (2006) 78:417–24. 10.1002/jmv.20556 16482545 PMC7166776

[27] Yokota S Yokosawa N Okabayashi T Suzutani T Miura S Jimbow K. Induction of suppressor of cytokine signaling-3 by herpes simplex virus type 1 contributes to inhibition of the interferon signaling pathway. J Virol. (2004) 78:6282–6. 10.1128/JVI.78.12.6282-6286.2004 15163721 PMC416529

[28] Akhtar LN Qin H Muldowney MT Yanagisawa LL Kutsch O Clements JE. Suppressor of cytokine signaling 3 inhibits antiviral IFN-beta signaling to enhance HIV-1 replication in macrophages. J Immunol. (2010) 185:2393–404. 10.4049/jimmunol.0903563 20631305 PMC3935802

[29] Persico M Capasso M Persico E Svelto M Russo R Spano. Suppressor of cytokine signaling 3 (SOCS3) expression and hepatitis C virus-related chronic hepatitis: insulin resistance and response to antiviral therapy. Hepatology. (2007) 46:1009–15. 10.1002/hep.21782 17668875

[30] Koeberlein B zur Hausen A Bektas N Zentgraf H Chin R Nguyen LT. Hepatitis B virus overexpresses suppressor of cytokine signaling-3 (SOCS3) thereby contributing to severity of inflammation in the liver. Virus Res. (2010) 148:51–9. 10.1016/j.virusres.2009.12.003 20005910

[31] Zheng J Yang P Tang Y Pan Z Zhao D. Respiratory syncytial virus nonstructural proteins upregulate SOCS1 and SOCS3 in the different manner from endogenous IFN signaling. J Immunol Res. (2015) 2015:738547. 10.1155/2015/738547 26557722 PMC4628668

[32] Zhang L Badgwell DB Bevers JJ III Schlessinger K Murray PJ. IL-6 signaling via the STAT3/SOCS3 pathway: functional analysis of the conserved STAT3 N-domain. Mol Cell Biochem. (2006) 288:179–89. 10.1007/s11010-006-9137-3 16718380 PMC2441693

[33] Gatto L Berlato C Poli V Tininini S Kinjyo I Yoshimura A. Analysis of SOCS-3 promoter responses to interferon gamma. J Biol Chem. (2004) 279:13746–54. 10.1074/jbc.M308999200 14742442

[34] Mosser DM Zhang X. Interleukin-10: new perspectives on an old cytokine. Immunol Rev. (2008) 226:205–18. 10.1111/j.1600-065X.2008.00706.x 19161426 PMC2724982

[35] Wang S Li H Chen Y Wei H Gao GF Liu H. Transport of influenza virus neuraminidase (NA) to host cell surface is regulated by ARHGAP21 and Cdc42 proteins. J Biol Chem. (2012) 287:9804–16. 10.1074/jbc.M111.312959 22318733 PMC3323004

[36] Ouyang J Zhu X Chen Y Wei H Chen Q Chi X. NRAV, a long noncoding RNA, modulates antiviral responses through suppression of interferon-stimulated gene transcription. Cell host Microbe. (2014) 16:616–26. 10.1016/j.chom.2014.10.001 25525793 PMC7104942

[37] Koskela HL Eldfors S Ellonen P van Adrichem AJ Kuusanmaki H Andersson EI. Somatic STAT3 mutations in large granular lymphocytic leukemia. N Engl J Med. (2012) 366:1905–13. 10.1056/NEJMoa1114885 22591296 PMC3693860

[38] Li F Chen Y Zhang Y Ouyang J Wang Y Yan R. Robust expression of vault RNAs induced by influenza A virus plays a critical role in suppression of PKR-mediated innate immunity. Nucleic Acids Res. (2015) 43:10321–37. 10.1093/nar/gkv1078 26490959 PMC4666359

[39] Chen Y Ouyang J Yan R Maarouf MH Wang X Chen B. Silencing SOCS3 markedly deteriorates spondyloarthritis in mice induced by minicircle DNA expressing IL23. Front Immunol. (2018) 9:2641. 10.3389/fimmu.2018.02641 30487798 PMC6246747

[40] Maarouf M Chen B Chen Y Wang X Rai KR Zhao Z. Identification of lncRNA-155 encoded by MIR155HG as a novel regulator of innate immunity against influenza A virus infection. Cell Microbiol. (2019) 21:e13036. 10.1111/cmi.13036 31045320

[41] da Silva CG Studer P Skroch M Mahiou J Minussi DC Peterson CR. A20 promotes liver regeneration by decreasing SOCS3 expression to enhance IL-6/STAT3 proliferative signals. Hepatology. (2013) 57:2014–25. 10.1002/hep.26197 23238769 PMC3626749

[42] Baker BJ Qin H Benveniste EN. Molecular basis of oncostatin M-induced SOCS-3 expression in astrocytes. Glia. (2008) 56:1250–62. 10.1002/glia.20694 18571793 PMC2621074

[43] Choe JY Park KY Park SH Lee SI Kim SK. Regulatory effect of calcineurin inhibitor, tacrolimus, on IL-6/sIL-6R-mediated RANKL expression through JAK2-STAT3-SOCS3 signaling pathway in fibroblast-like synoviocytes. Arthritis Res Ther. (2013) 15:R26. 10.1186/ar4162 23406906 PMC3672788

[44] Rottenberg ME Carow B. SOCS3 and STAT3, major controllers of the outcome of infection with *Mycobacterium tuberculosis*. Semin Immunol. (2014) 26:518–32. 10.1016/j.smim.2014.10.004 25458989

[45] Ma X Reynolds SL Baker BJ Li X Benveniste EN Qin H. IL-17 enhancement of the IL-6 signaling cascade in astrocytes. J Immunol. (2010) 184:4898–4906. 10.4049/jimmunol.1000142 20351184 PMC3769161

[46] Shi D Yang J Wang Q Li D Zheng H Mei H. SOCS3 ablation enhances DC-derived Th17 immune response against candida albicans by activating IL-6/STAT3 *in vitro*. Life Sci. (2019) 222:183–194. 10.1016/j.lfs.2019.03.009 30851337

[47] Yu Z Zhang W Kone BC. Signal transducers and activators of transcription 3 (STAT3) inhibits transcription of the inducible nitric oxide synthase gene by interacting with nuclear factor kappaB. Biochem J. (2002) 367:97–105. 10.1042/bj20020588 12057007 PMC1222853

[48] Svitek N Rudd PA Obojes K Pillet S von Messling V. Severe seasonal influenza in ferrets correlates with reduced interferon and increased IL-6 induction. Virology. (2008) 376:53–9. 10.1016/j.virol.2008.02.035 18420248

[49] Chen JL Yan R Ouyang J Chen B Zeng X Inflenza A virus-induced IL-6 storm is regulated by SOCS3. Third International Congress on Microbiology and Pharmaceutical Microbiology and Annual Summit on Sexual and Reproductive Health, Atlanta, USA. 02-03 October, 2017. J sex Reprod med. (2017) 1:45 Available online at: https://www.pulsus.com/conference-abstracts-files/microbiology-reproductive-health-2017-posters-abstracts.digital/files/assets/common/downloads/microbiology-reproductive-health-2017-posters-abstracts.pdf

[50] Hunter CA Jones SA. IL-6 as a keystone cytokine in health and disease. Nat Immunol. (2015) 16:448–57. 10.1038/ni.3153 25898198

[51] Garcia-Sastre A Longhi MP Wright K Lauder SN Nowell MA Jones GW Interleukin-6 is crucial for recall of influenza-specific memory CD4+ T cells. PLoS Pathog. (2008) 4:1000006 10.1371/journal.ppat.1000006 PMC2279258 18389078

[52] Sun K Salmon S Yajjala VK Bauer C Metzger DW. Expression of suppressor of cytokine signaling 1 (SOCS1) impairs viral clearance and exacerbates lung injury during influenza infection. PLoS Pathog. (2014) 10:e1004560. 10.1371/journal.ppat.1004560 25500584 PMC4263766

[53] Lang R Pauleau AL Parganas E Takahashi Y Mages J Ihle JN. SOCS3 regulates the plasticity of gp130 signaling. Nat Immunol. (2003) 4:546–50. 10.1038/ni932 12754506

[54] Feeley EM Sims JS John SP Chin CR Pertel T Chen LM. IFITM3 inhibits influenza A virus infection by preventing cytosolic entry. PLoS Pathog. (2011) 7:e1002337. 10.1371/journal.ppat.1002337 22046135 PMC3203188

[55] Everitt AR Clare S Pertel T John SP Wash RS Smith SE. IFITM3 restricts the morbidity and mortality associated with influenza. Nature. (2012) 484:519–23. 10.1038/nature10921 22446628 PMC3648786

[56] Wang S Chi X Wei H Chen Y Chen Z Huang S. Influenza A virus-induced degradation of eukaryotic translation initiation factor 4B contributes to viral replication by suppressing IFITM3 protein expression. J Virol. (2014) 88:8375–85. 10.1128/JVI.00126-14 24829357 PMC4135930

[57] Arnold CE Whyte CS Gordon P Barker RN Rees AJ Wilson HM. A critical role for suppressor of cytokine signalling 3 in promoting M1 macrophage activation and function *in vitro* and *in vivo*. Immunology. (2014) 141:96–110. 10.1111/imm.12173 24088176 PMC3893853

[58] Spangenburg EE Brown DA Johnson MS Moore RL. Exercise increases SOCS-3 expression in rat skeletal muscle: potential relationship to IL-6 expression. J Physiol. (2006) 572:839–48. 10.1113/jphysiol.2005.104315 16484300 PMC1780003

[59] Park SH Kim KE Hwang HY Kim TY. Regulatory effect of SOCS on NF-kappaB activity in murine monocytes/macrophages. DNA cell biol. (2003) 22:131–9. 10.1089/104454903321515931 12713738

[60] Grivennikov SI Karin M. Dangerous liaisons: STAT3 and NF-kappaB collaboration and crosstalk in cancer. Cytokine Growth Factor Rev. (2010) 21:11–9. 10.1016/j.cytogfr.2009.11.005 20018552 PMC2834864

[61] Lee H Deng J Xin H Liu Y Pardoll D Yu H. A requirement of STAT3 DNA binding precludes Th-1 immunostimulatory gene expression by NF-κB in tumors. Cancer Res. (2011) 71:3772–80. 10.1158/0008-5472.CAN-10-3304 21502401 PMC3107358

[62] Folch-Puy E Granell S Dagorn JC Iovanna JL Closa D Pancreatitis-associated protein I suppresses NF-kappaB activation through a JAK/STAT-mediated mechanism in epithelial cells. J Immunol. (2006) 176:3774–9. 10.4049/jimmunol.176.6.3774 16517747

[63] Dhar K Rakesh K Pankajakshan D Agrawal DK. SOCS3 promotor hypermethylation and STAT3-NF-κB interaction downregulate SOCS3 expression in human coronary artery smooth muscle cells. Am J Physiol Heart Circ Physiol. (2013) 304:H776–85. 10.1152/ajpheart.00570.2012 23335796 PMC3602771

[64] Yang J Liao X Agarwal MK Barnes L Auron PE Stark GR. Unphosphorylated STAT3 accumulates in response to IL-6 and activates transcription by binding to NFkappaB. Genes Dev. (2007) 21:1396–408. 10.1101/gad.1553707 17510282 PMC1877751

