# Supplementary material for: Influenza Virus-Induced Robust Expression of SOCS3 Contributes to Excessive Production of IL-6
Source: Front Immunol. 2019 Aug 16;10:1843. doi: 10.3389/fimmu.2019.01843 (PMC6706793; doi:10.3389/fimmu.2019.01843)
Supplement: Supplementary file 1 [file Data_Sheet_1.pdf]

SUPPLEMENTAL MATERIAL

TABLES

Table 1. Sequences of primers used in this study.

Name	Sequence (5'-3')
SOCS3 (human) forward	GACGGAGACTTCGATTCGGG
SOCS3 (human) reverse	GCTGGTACTCGCTCTTGGAG
IFN β (human) forward	GCTCTCCTGTTGTGCTTCTCCAC
IFN β (human) reverse	CAATAGTCTCATTCCAGCCAGTGC
IL-6 (human) forward	AATGAGGAGACTTGCCTGGTG
IL-6 (human) reverse	TGAGGTGCCCATGCTACATT
IL-28 (human) forward	AGCTGCAGGCCTTTAAGAGG
IL-28 (human) reverse	TCCAGAACCTTCAGCGTCAG
IL-6Rα (human) forward	TCACTGTGTCATCCACGACG
IL-6Rα (human) reverse	CTGGATTCTGTCCAAGGCGT
gp130 (human) forward	CAGTGGTCACCTCACACTCC
gp130 (human) reverse	GTAGATCTTCTGGCCGCTCC
IL-1β (human) forward	CAGAAGTACCTGAGCTCGCC
IL-1β (human) reverse	TCGTGCACATAAGCCTCGTT
TNFα (human) forward	ACCCATGTACTCCTCACCCA

TNFα (human) reverse	CTCACAGGGCAATGATCCCA
wTG forward	AAATCCTGGTTGCTGTCTCTTTATG
wTG reverse	GGAAGGTCCGCTGGATTGA
GFP forward	CGTCCAGGAGCGCACCATCTTCTT
GFP reverse	ATCGCGCTTCTCGTTGGGGTCTTT
NP (CA04) forward	GTGGTCAGCCTGATGAGACC
NP (CA04) reverse	TCCGTCCTTCATTGTTCCCG
NP forward	TCAAACGTGGGATCAATG
NP reverse	GTGCAGACCGTGCTAGAA
SOCS3 (mouse) forward	GGAGATTTCGCTTCGGGACT
SOCS3 (mouse) reverse	TCGCTTTTGGAGCTGAAGGT
IL-6 (mouse) forward	GGGACTGATGCTGGTGACAA
IL-6 (mouse) reverse	CGCACTAGGTTTGCCGAGTA
ACTIN (mouse) forward	GCTGCCTCAACACCTCAACCC
ACTIN (mouse) reverse	GTCCCTCACCCTCCCAAAAG
IL-1β (mouse) forward	GTGGCAGCTACCTGTGTCTT
IL-1β (mouse) reverse	GGAGCCTGTAGTGCAGTTGT
TNFα (mouse) forward	ACCCTCACACTCACAAACCA
TNFα (mouse) reverse	ACCCTGAGCCATAATCCCCT

Fig. S1

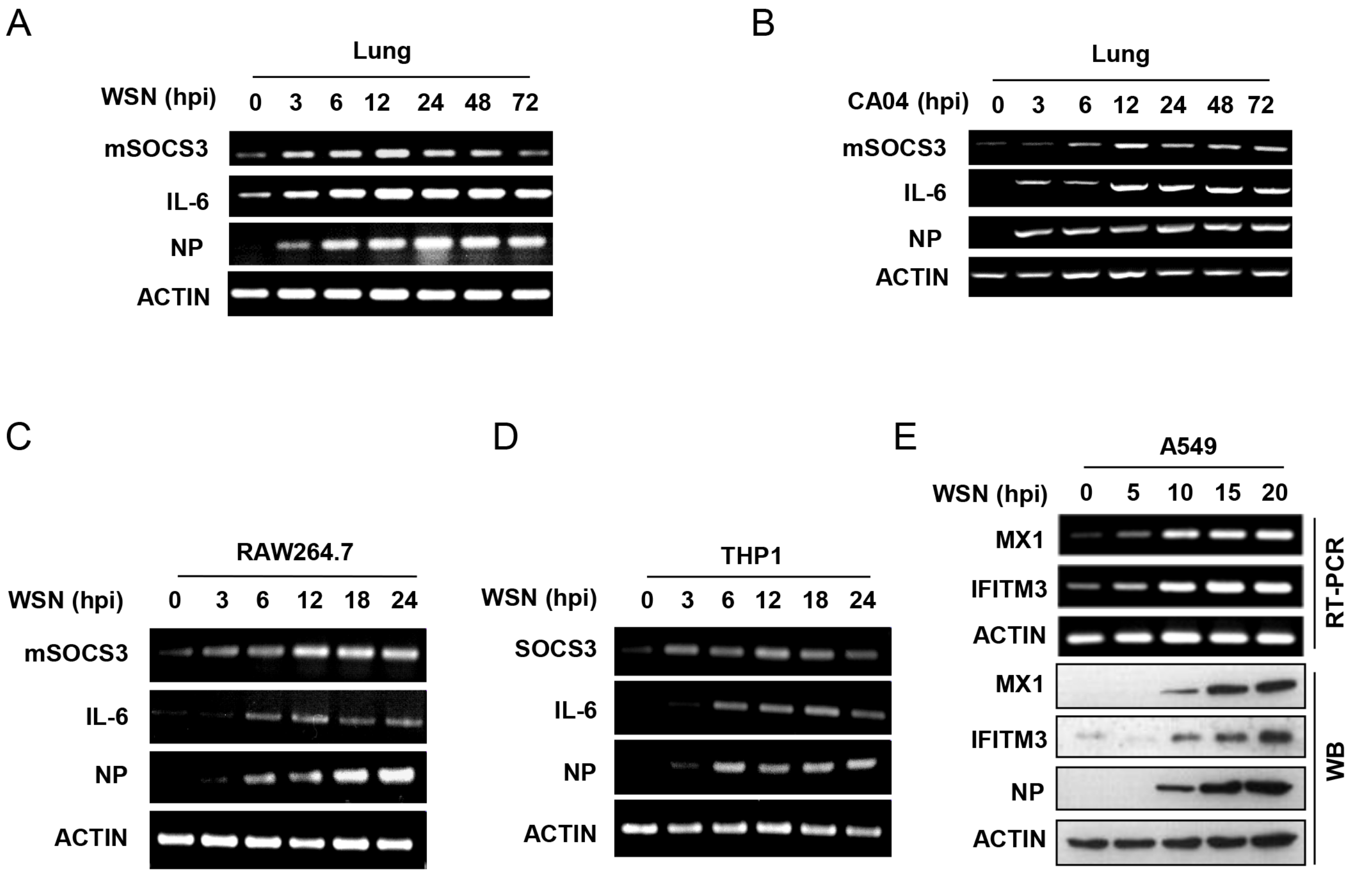
A
Lung
WSN (hpi) 0 3 6 12 24 48 72
mSOCS3
IL-6
NP
ACTIN
B
Lung
CA04 (hpi) 0 3 6 12 24 48 72
mSOCS3
IL-6
NP
ACTIN
C
RAW264.7
WSN (hpi) 0 3 6 12 18 24
mSOCS3
IL-6
NP
ACTIN
D
THP1
WSN (hpi) 0 3 6 12 18 24
SOCS3
IL-6
NP
ACTIN
E
A549
WSN (hpi) 0 5 10 15 20
MX1
IFITM3
ACTIN
RT-PCR
MX1
IFITM3
NP
ACTIN
WB

Fig. S1 IAV infection induces robust expression of SOCS3 and IL-6 *in vitro* and *in vivo*. (A, B) C57BL/6 mice were intranasally infected with WSN (A), CA04 (B) (5×104 PFU) and were sacrificed at the indicated times. The mSOCS3 and IL-6 mRNA levels in lungs were determined via RT-PCR. Shown are representative blots from three independent experiments (C, D) RAW264.7 (C) or THP1 (D) cells were infected with WSN for the indicated time. The mRNA levels of SOCS3, IL-6, gp130, IL-6Rα were measured by RT-PCR. Shown are representative data from three independent experiments. (E) A549 cells were infected with WSN for the indicated times, the expression of MX1 and IFITM3 were examined by RT-PCR or Western blotting. Shown are representative data from three independent experiments.

Fig. S2

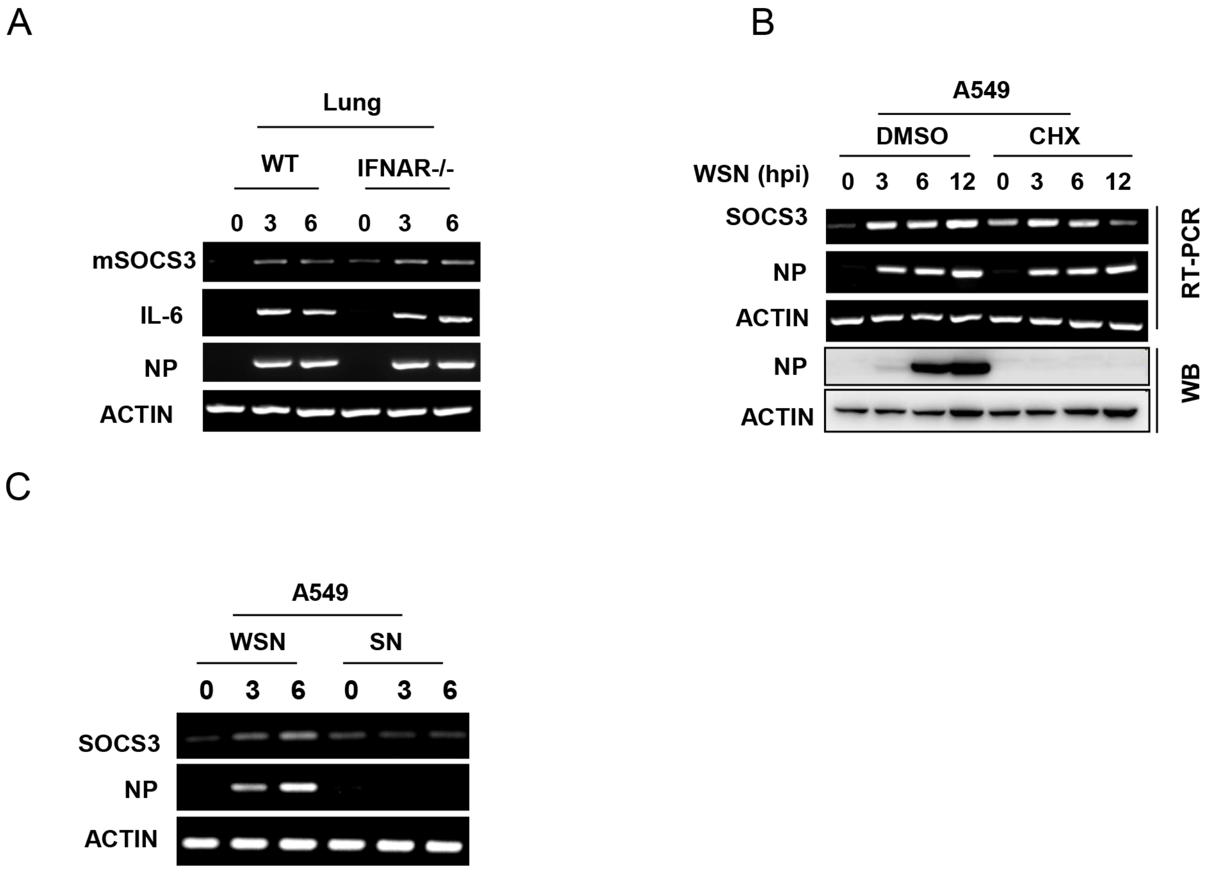

A
Lung
WT
IFNAR-/-
0 3 6 0 3 6
mSOCS3
IL-6
NP
ACTIN
B
A549
DMSO
CHX
WSN (hpi)
0 3 6 12 0 3 6 12
SOCS3
NP
ACTIN
RT-PCR
NP
ACTIN
WB
C
A549
WSN
SN
0 3 6 0 3 6
SOCS3
NP
ACTIN

Fig. S2 Expression of SOCS3 at early stage of IAV infection is independent of induction of cytokines including IL-6. (A) Shown are mRNA levels of mSOCS3 and IL-6 determined via RT-PCR in lung tissues from IFNAR$^{-/-}$ mice and WT littermates after WSN infection for the indicated time. Shown are representative data from three independent experiments. (B) A549 cells were treated with DMSO (0.1 %) or CHX (1 ng/ml) for 30 min, and then infected with WSN (MOI = 1) for indicated time. The expression of SOCS3 was determined by RT-PCR. (C) A549 cells were infected by WSN for indicated time. Supernatants (SN) derived from these cell cultures were used to stimulate the native A549 for 1 h. Both infected cells and SN-stimulated cells were used to analyze SOCS3 expression via RT-PCR. Shown are representative blots from three independent experiments.

Fig. S3

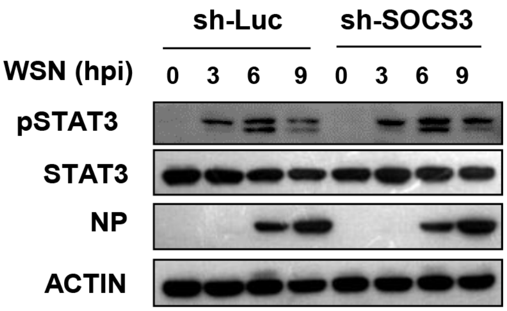

Fig. S3 IAV impairs IL-6-stimulated STAT3 activation by upregulation of SOCS3. A549 cells expressing sh-SOCS3 RNA or control sh-Luc were infected without or with WSN virus for the indicated time. The phosphorylation of STAT3 were examined by Western blotting. Shown are representative data from three independent experiments.

Fig. S4

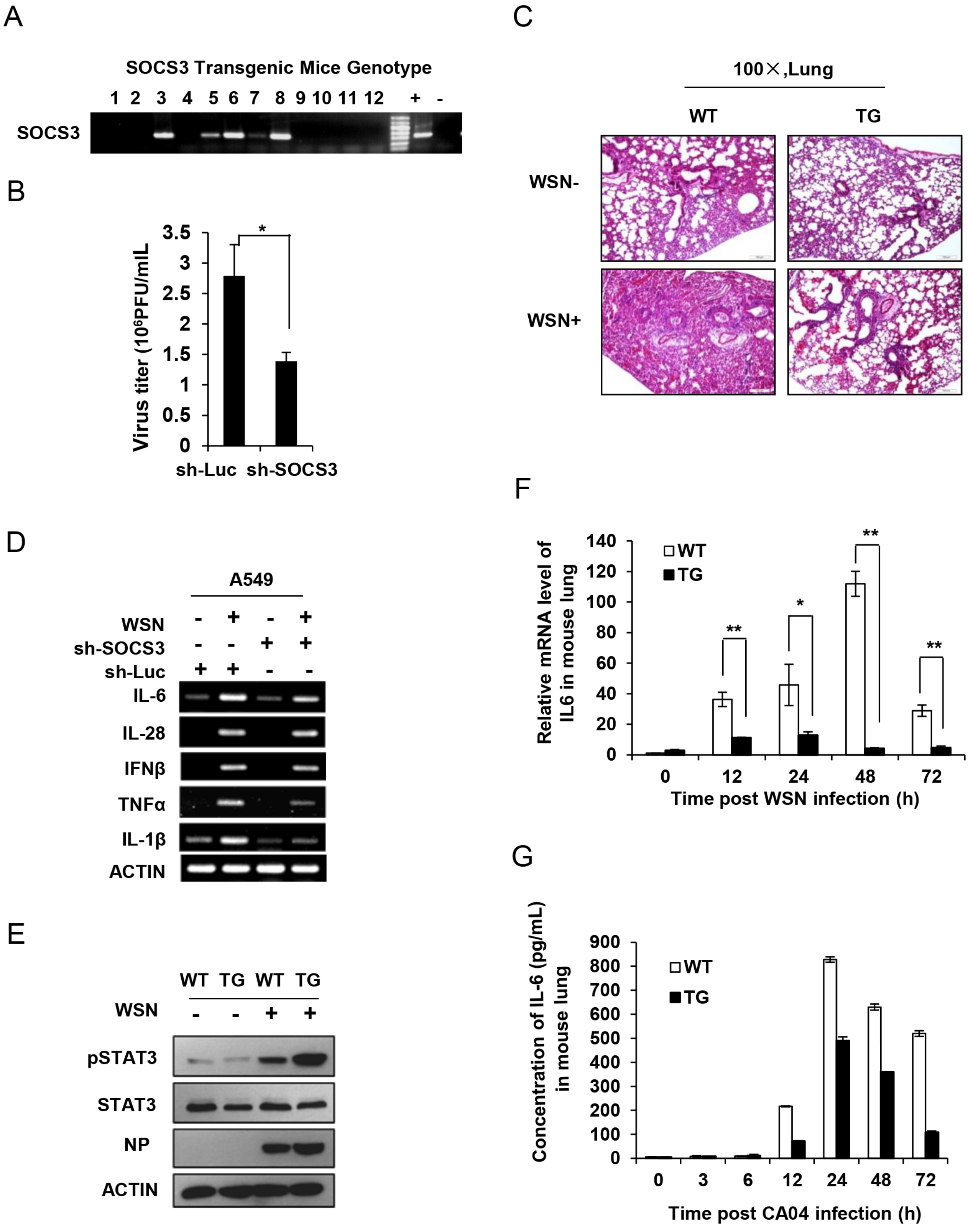
A
SOCS3 Transgenic Mice Genotype
1 2 3 4 5 6 7 8 9 10 11 12 + -
SOCS3
B
Virus titer (10⁶PFU/mlL
3.5
3
2.5
2
1.5
1
0.5
0
*
sh-Luc
sh-SOCS3
C
100×,Lung
WT
TG
WSN-
WSN+
D
A549
WSN
sh-SOCS3
sh-Luc
IL-6
IL-28
IFNβ
TNFα
IL-1β
ACTIN
E
WT TG WT TG
WSN
pSTAT3
STAT3
NP
ACTIN
F
Relative mRNA level of IL6 in mouse lung
WT
TG
140
120
100
80
60
40
20
0
12
24
48
72
Time post WSN infection (h)
G
Concentration of IL-6 (pg/mL) in mouse lung
WT
TG
900
800
700
600
500
400
300
200
100
3
6
Time post CA04 infection (h)

Fig. S4 Targeted disruption of SOCS3 expression decreases viral replication during IAV infection *in vitro* and *in vivo*. (A) The SOCS3-knockdown transgenic mice were genotyped via PCR. Shown is representative genotyping results of SOCS3-knockdown transgenic mice. Numbers 1-12, individual mice; +, positive control; -, negative control. (B) A549 cells stably expressing the shRNAs targeting SOCS3 or luciferase were infected with WSN (MOI=0.3). The viral titers in cell culture supernatants were determined by plaque forming assay (16 hpi). Data are shown as mean ± S.D.; n=3; *, $P < 0.05$ (Student's T test). (C) WT mice and SOCS3-knockdown TG mice were infected with WSN for 3 days. Shown are representative photomicrographs of the mouse lungs stained with hematoxylin and eosin (HE). The pathological changes of lungs in WT mice were more serious than that of the TG counterparts. Bars, 100 μm. (D) SOCS3-knockdown or control A549 cells were infected with or without WSN (MOI = 0.3) for 15 h and the mRNA levels of IL-6, IL-1β, TNFα, IFNβ, IL-28A/B and were examined by RT-PCR. (E-G) TG mice and WT mice were intranasally infected with WSN (E, F) or CA04 (G) for 24 h (E) or indicated time points (F, G). The levels of phosphorylated STAT3 in mouse lungs were determined by Western blotting (E) and the mRNA or protein levels of IL-6 were examined by quantitative RT-PCR (RT-qPCR) (F) or ELISA (G), respectively. Shown are representative data from three independent experiments. Data are shown as mean ± S.D.; *, $P < 0.05$; **, $P < 0.01$ (Student's T test).

Fig. S5

A

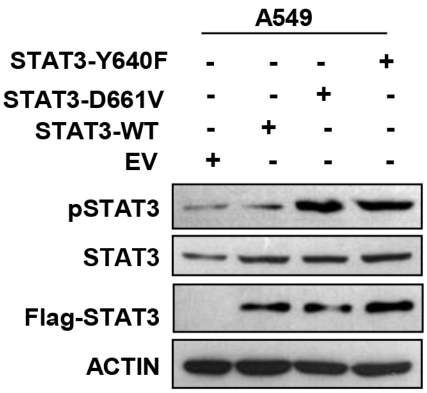

A549
STAT3-Y640F - - - +
STAT3-D661V - - + -
STAT3-WT - + - -
EV + - - -
pSTAT3
STAT3
Flag-STAT3
ACTIN

B

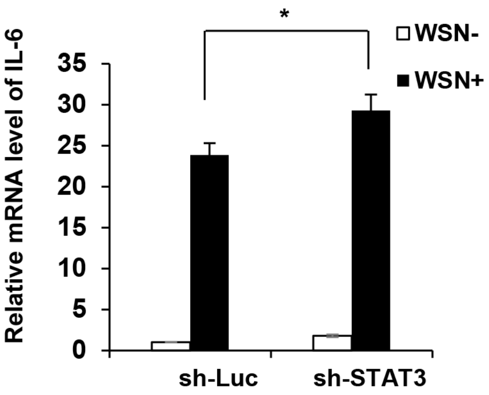

*
WSN-
WSN+
Relative mRNA level of IL-6
35
30
25
20
15
10
5
0
sh-Luc
sh-STAT3

Fig. S5 Altering STAT3 activity has significant effect on IL-6 expression. (A) The pSTAT3 levels in A549 cells stably expressing STAT3-D661V, STAT3-Y640F, STAT3-WT or control (empty vector, EV) were detected by Western blotting. Shown are representative data from three independent experiments. (B) A549 cells expressing shRNAs targeting STAT3 or luciferase (Luc) were infected with or without WSN, the IL-6 mRNA levels were examined via RT-qPCR. Shown are representative blots from three independent experiments. Data are shown as mean ± S.D.; *, $P < 0.05$;
